# Predictors of ICS/LABA prescribing in COPD patients: a study from general practice

**DOI:** 10.1186/1471-2296-15-42

**Published:** 2014-03-05

**Authors:** Elin Drivenes, Anders Østrem, Hasse Melbye

**Affiliations:** 1 General Practice Research Unit, Department of Community Medicine, University of Tromsø, Wititten, Norway; 2 General Practice Research Unit, Department of Health and Society, University of Oslo, Oslo, Norway

**Keywords:** Prescription, Adrenergic beta_2_ agonists, Corticosteroids, COPD, Primary care

## Abstract

**Background:**

A combination of inhaled corticosteroid and long-acting beta_2_ agonist (ICS/LABA) is used frequently to treat chronic obstructive pulmonary disease (COPD) patients. The aim of the study was to determine whether prescribing ICS/LABA to COPD patients in primary care in 2009/10 was within the GOLD guidelines and whether and to what degree patient characteristics were associated with prescription of these drugs by GPs.

**Methods:**

This was a cross-sectional study in seven Norwegian GP practices. Patients registered with a diagnosis of asthma or COPD in the previous five years were included.

**Results:**

Among the 376 patients included in the analysis, 149 patients had COPD, defined as a post-bronchodilator FEV_1_/FVC <0.7 and 55.6% of these patients were treated with ICS/LABA. The rate of prescribing was significantly higher in the COPD patients also diagnosed with asthma than in those with COPD as the only diagnosis, 66.7%, and 39.0%, respectively (*P* = 0.001). The prescribing rate in the latter subgroup would have been 18.6% if the 2007 GOLD guidelines had been followed. One or more exacerbations in the previous year was the strongest predictor of ICS/LABA prescribing in the COPD patients who were not registered with a concomitant diagnosis of asthma (OR 3.2, 95% CI 1.0–10.0) but this association was limited to the patients with severe disease (FEV_1_% predicted <50) (OR 13.5, 95% CI 1.8–101.1). Cardiovascular disease was associated with decreased ICS/LABA prescribing (OR 0.4, 95% CI 0.2–0.8) in the COPD group. A Kappa coefficient of 0.32 was found between the actual prescribing rate and that recommended in the 2007 GOLD guidelines.

**Conclusions:**

Overprescribing of ICS/LABA for the COPD patients was shown. Previous exacerbation was a strong predictor of ICS/LABA prescribing only in patients with severe COPD. Because of the low emphasis on previous exacerbation when prescribing for COPD patients with mild to moderate disease, the actual prescribing rate agreed more closely with the GOLD guidelines from 2007 than with those published in 2011. Cardiovascular disease was associated with decreased prescribing, indicating that GPs adjust the treatment in cases with multimorbidity.

## Background

Although smoking cessation remains the most important treatment of chronic obstructive lung disease (COPD) [1], there is evidence that pharmacotherapy also is important for decreasing symptoms and exacerbation [2]. Inhaled corticosteroid (ICS) in combination with long-acting beta_2_ agonists (LABAs) is a very successful option in patients with asthma [3]. Although less efficacious in COPD patients, ICS/LABA has become a common choice for COPD patients based on the findings of studies showing its efficacy in reducing the frequency and severity of exacerbation [2]. The evidence for the usefulness of ICS/LABA for treating moderate COPD has been limited. The Global Initiative for Chronic Obstructive Lung Diseases (GOLD) guidelines between 2007 and 2010 recommended such treatment only for patients with severe COPD, defined as GOLD III–IV, forced expiratory volume in one second (FEV_1_) <50% of the predicted value, and frequent (≥2 yearly) exacerbation [4]. ICS/LABA is prescribed for most COPD patients in some countries and to patients with FEV_1_% predicted ≥50 [5,6]. A more positive attitude towards the use of ICS/LABA in patients with moderate COPD is reflected in the 2011 GOLD guidelines, which say that such treatment should now be considered for mild to moderate COPD when the patient experiences frequent exacerbations.

The aim of this study was to describe the prescribing of ICS/LABA to patients with COPD aged 40 years or more in Norwegian general practice in 2009/2010 and to determine whether and to what extent the pattern of prescription corresponded to the GOLD guidelines for that period. We also wanted to determine to which degree various patient characteristics were associated with the prescribing by GPs and, in particular, whether previous exacerbation could predict ICS/LABA prescribing.

## Methods

General practitioners (GPs) from seven Norwegian practices were asked to participate in the study. Only practices with an electronic medical record system compatible with the registration software used were selected. These practices had 43,241 patients listed, of whom 18,931 (43.8%) were aged 40 years or older. Of these, 1,784 (9.4%) had been registered with a diagnosis of asthma, COPD, or both in the previous five years. A random sample of these 1,784 patients, following an alphabetical order, was invited by mail to participate in the study. The patients gave written informed consent and were examined during a stable phase of their disease between April 2009 and March 2010.

### Registrations

Asthma and COPD diagnoses, recorded by a GP in the electronic medical record during the five years before the examination, were automatically registered by software developed by Mediata AS, Norway. Comorbidities were registered by the GPs on a computerized questionnaire linked to the medical record. The GPs also registered the medications currently prescribed for obstructive lung disease. Exacerbation of asthma or COPD treated with antibiotics and/or oral corticosteroids in the previous 12 months were also recorded. The patients recorded on a form whether they had been hospitalized for asthma or COPD in the previous 12 months, follow-up of their lung disease in secondary care, and their history of smoking. Symptoms and limitations of daily activities for the seven days preceding the examination were registered using the Clinical COPD Questionnaire (CCQ), yielding scores of 0–6 for 10 items [7]. The patients also reported the medications they had used the day before the examination. The patients were instructed not to take any pulmonary medication on the day of the examination.

### Spirometry

Spirometry was performed by trained health workers following the guidelines from European Respiratory Society and American Thoracic Society [8], and the Spirare SPS310 spirometer (Diagnostica AS, Norway) was used in all practices. The patients were seated, and a nose clip was not used. Post-bronchodilator spirometry was performed 20 min after inhalation of 0.4 mg salbutamol. The post-bronchodilator FEV_1_ and forced vital capacity (FVC) were used in the analyses. Norwegian reference values for spirometry were applied [9].

### Analysis

Patients with a post-bronchodilator FEV_1_/FVC ratio <0.7 were classified as having COPD, even though some had a combination of asthma and COPD [10]. Patients with spirometry incompatible with COPD according to the GOLD guidelines (FEV_1_/FVC ratio ≥0.7), including patients with a restrictive spirometry pattern (both FEV_1_% predicted and FVC% predicted <80) were analysed separately to compare the COPD patients with other patients deemed to have obstructive lung disease. In the analysis, FEV_1_% predicted was categorized into three levels: <50%, 50–80% and ≥80%. The CCQ score was also categorized into three levels: <1, 1–2, and ≥2.

Exacerbation of COPD was defined as in the ECLIPSE study as follows. “Events that led a care provider to prescribe antibiotics or corticosteroids (or both) or that led to hospitalization” [5]. One or more episodes of moderate to severe exacerbation [11] during the previous year were categorized as “exacerbation in the previous year”. Patients were classified as having cardiovascular disease if their GP had recorded coronary heart disease, other heart disease, or stroke. Treatment with both ICS and LABA was classified as treatment with ICS/LABA regardless of whether the medicine was given in a combination inhaler or in separate inhalers.

The significance of differences between groups were analysed by chi-square statistics. Agreement between actual and recommended prescribing was analysed by Kappa statistics. The category of exacerbation in the previous year was used as a proxy for frequent exacerbation in this analysis. Predictors of prescribing ICS/LABA were analysed by univariable logistic regression. SPSS 19.0 (IBM Corp., Armonk, NY, USA) was used for the statistical analyses.

The study was approved by the Regional Committee for Medical and Health Research Ethics.

## Results

### Patients’ characteristics

Of the random sample of 1,111 invited, 380 (34.2%) accepted and completed in the examination. Two patients were excluded from the analysis because of symptoms and signs of an ongoing exacerbation, and two patients were excluded because they had not completed post-bronchodilator spirometry. Of the 376 remaining patients, 74 (19.7%) had been registered with a diagnosis of COPD only in the previous five years, 210 (55.8%) with asthma only, and 92 (24.5%) with both diagnoses (Table 1). Among the 376 patients, 23.7% reported that their current diagnosis had been given by a secondary care doctor, and 7.2% that their lung disease was usually followed up at this level of care.

**Table 1:** Patient characteristics and diagnosis registered in the medical records for the previous five years in 376 patients aged ≥40 years diagnosed with an obstructive lung disease in primary care

	**FEV**_**1**_**/FVC <0.7 (n = 149)**		**FEV**_**1**_**/FVC ≥0.7 (n = 227)**		
	**n**	**(%)**	**n**	**(%)**	**P-value**
Men	69	46.3	74	32.6	0.007
Age ≥65 years	82	55.0	70	30.8	<0.001
Smoking					
Current	44	29.5	62	27.3	<0.001^a^
Former	86	57.7	87	38.3	
Never	19	12.8	78	34.4	
GP diagnosis					
COPD only	59	39.6	15	6.6	<0.001^a^
COPD and asthma	54	36.2	38	16.7	
Asthma only	36	24.4	174	76.7	
Other illness (GP reported)					
Allergic rhinitis and/or eczema	46	30.9	125	55.1	<0.001
Cardiovascular disease	64	43.0	58	25.6	<0.001
Exacerbation in the previous year	54	36.2	46	20.3	0.001
FEV_1_% predicted					
<50	44	29.5	7	3.1	<0.001^a^
50–80	87	58.4	77	33.9	
≥80	18	12.1	143	63.0	
CCQ mean score^b^					
<1	30	21.6	64	29.9	0.03^a^
1–2	50	36.0	81	37.9	
≥2	59	42.4	69	32.2	

^a^Chi-square trend.

^b^Data missing for 23 patients.

The median age was 62 years. The mean CCQ score was 1.68 (SD 0.98) for the 353 patients who answered all 10 CCQ questions. Post-bronchodilator spirometry indicating COPD (FEV_1_/FVC <0.7) was found in 149 patients (39.6%) and more frequently among men (48.3%) than among women (34.3%) (Table 1). Of these 149 patients, 70.5% were classified according to the 2007 GOLD stages with mild to moderate COPD (GOLD I–II) and 29.5% with severe or very severe COPD (GOLD III–IV); 39.6% had been given a COPD diagnosis but no asthma diagnosis by their GP, and 36.2% had been diagnosed with both asthma and COPD (Table 1). Exacerbation in the previous year was registered in 54 of the 149 COPD patients (36.2%) and in 19 (32.2%) of the 59 COPD patients with COPD as the only diagnosis registered. A combination of FEV_1_% predicted <50 and exacerbation in the previous year, indicating that ICS/LABA could be prescribed according to the 2007 GOLD guidelines, was found in 19 (12.6%) of the 149 COPD patients and in 11 (18.6%) of the 59 COPD patients with COPD as the only diagnosis.

### Medication prescribed

**Figure 1:**
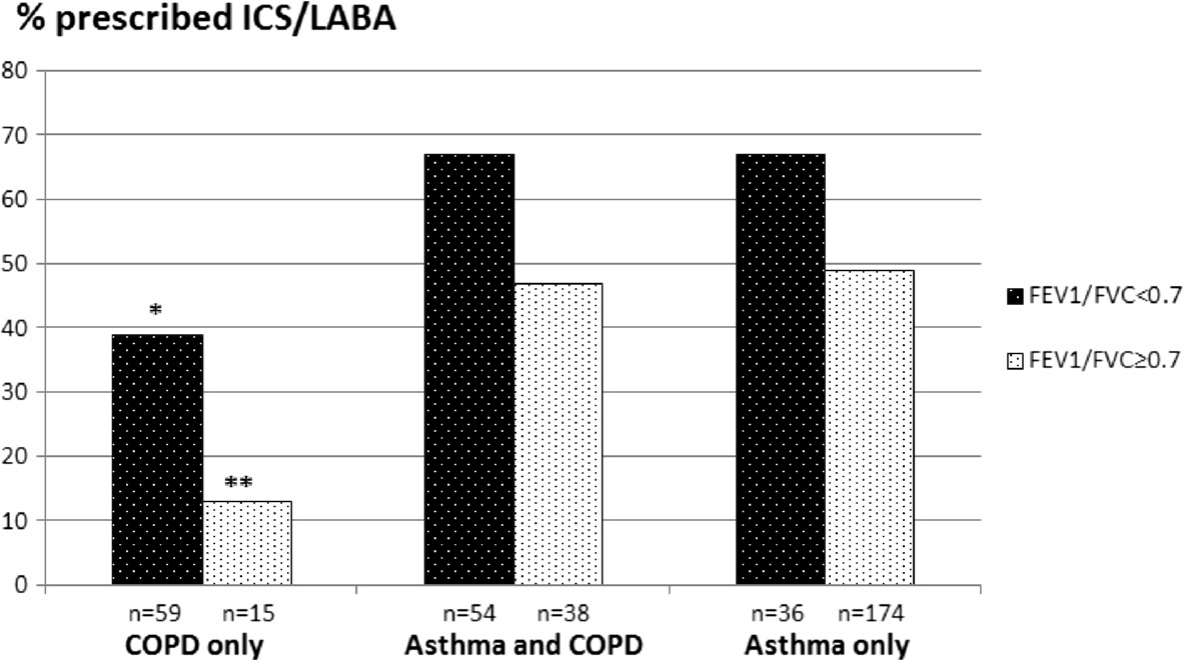
Frequency of prescribing ICS/LABA to patients with asthma or COPD in primary care according to lung function and diagnosis registered by GPs.

Two hundred and ninety-five (78.5%) patients were on pulmonary medication. ICS/LABA was prescribed to 55.7% of all patients with COPD, and in 94% of these as a combined inhaler. ICS/LABA was prescribed (in a combined or separate inhaler) somewhat less frequently in those with an FEV_1_/FVC ≥0.7, but the difference was not significant (Table 2). ICS/LABA was prescribed to 63.6% of the patients with GOLD III–IV severity and to 52.4% of the patients with GOLD I–II (the difference was not significant). In the COPD group, the patients who had been diagnosed with asthma were prescribed ICS/LABA more frequently (66.7%) than were those who had COPD as the only diagnosis (39.0%) (*P* < 0.001) (Figure 1). The Kappa coefficient was 0.37 (SE 0.12) for the comparison between ICS/LABA prescribed to the COPD patients without a diagnosis of asthma and the medication recommended in the 2007 GOLD guidelines. Overprescribing was much more common than underprescribing (Table 3). The opposite was the case when comparing the actual prescribing with the recommended prescribing based on the 2011 GOLD guidelines, which produced a lower Kappa coefficient (Table 4).

**Table 2:** **Medication prescribed and medication taken on the previous day in 376 patients aged** ≥**40 years diagnosed with an obstructive lung disease in primary care according to whether COPD was indicated by spirometry (FEV**_**1**_**/FVC <0.7)**

	**FEV**_**1**_**/FVC <0.7 (n = 149)**		**FEV**_**1**_**/FVC ≥0.7 (n = 227)**	
**Medication**	**Prescribed %**	**Taken the previous day %**	**Prescribed %**	**Taken the previous day %**
SABA	42.3	24.8	35.7	19.8
LABA, no ICS	10.7	7.4	4.8	3.1
ICS, no LABA	8.1	6.0	14.5	10.6
ICS/LABA combined	55.7	49.0	46.7	37.4
Anticholinergics	41.6^a^	38.3^a^	10.3	5.7
Theophylline	1.3	0.75	1.3	0.9
Montelukast	7.3	5.4	13.2	10.0
Prednisolone	2.0	1.3	0	0.9
Any pulmonary medication	83.2	72.5^b^	75.3	62.6

^a^*P* < 0.001, difference between the two patient groups.

^b^*P* = 0.05, difference between the two patient groups.

SABA: short-acting beta_2_ agonist, LABA: long-acting beta_2_ agonist, ICS: inhaled corticosteroid.

**Table 3:** **Agreement between actual prescribing of ICS/LABA for 59 COPD patients (FEV**_**1**_**/FVC <0.7) with no concomitant diagnosis of asthma in 2009/10 and the prescribing recommended based on the 2007–2010 GOLD guidelines**

	**ICS/LABA recommended (FEV**_**1**_**% predicted <50 and previous exacerbation)**		
ICS/LABA	Yes	No	
Prescribed 2009/10			
Yes	9	14	23
No	2	34	36
	11	48	59
	Kappa = 0.37 (SE 0.12)		

**Table 4:** **Agreement between actual prescribing of ICS/LABA for 59 COPD patients (FEV**_**1**_**/FVC < 0.7) with no concomitant diagnosis of asthma in 2009/10 and the prescribing recommended based on the 2011 GOLD guidelines**

	**ICS/LABA recommended (FEV**_**1**_**% predicted < 50 or previous exacerbation)**		
ICS/LABA	Yes	No	
prescribed 2009/10			
Yes	14	9	23
No	17	19	36
	31^a^	28	59
	Kappa = 0.13 (SE 0.12)		

^a^Frequent exacerbation only was recorded in eight patients, predicted FEV_1_% <50% only in 12 patients, and both findings in 11 patients.

### Medication taken

Among the patients who took prescribed pulmonary medication, the great majority had taken prescribed medication on the previous day (Table 2). Among the COPD patients who had been prescribed pulmonary medication, 86.7% had taken ICS/LABA and 88.7% had taken an anticholinergic on the previous day. The corresponding frequencies among the patients with an FEV_1_/FVC ≥0.7 were 80.2% and 56.5%.

### Predictors of ICS/LABA prescribing

The strongest predictors of increased ICS/LABA prescribing in the COPD group were an asthma diagnosis registered by the GP and exacerbation registered in the previous year (Table 5). The same variables and increased mean CCQ score were significant predictors in the group with an FEV_1_/FVC ≥0.7. Cardiovascular disease was associated with a decreased ICS/LABA prescribing in the COPD group as was current smoking (*P* = 0.06). When evaluating the predictors in the 59 COPD patients who had not received an asthma diagnosis, previous exacerbation was the only significant predictor (Odds ratio (OR) 3.2, 95% confidence interval (CI) 1.0–10.0). When splitting these 59 patients into two groups according to FEV_1_% predicted, <50 and ≥50, previous exacerbation was a significant predictor when FEV_1_% predicted was <50 (OR 13.5, 95% CI 1.8–101.1) but not when FEV_1_% predicted was ≥50 (OR 0.7, 95% CI 0.1–4.2). Cardiovascular disease had the same OR (0.4) in the COPD patients without an asthma diagnosis as in all patients with an FEV_1_/FVC <0.7, but this association was not significant (*P* = 0.08).

**Table 5:** **Predictors of ICS/LABA prescribing as determined by bivariate logistic regression according to lung function in 376 patients aged ≥40 years diagnosed with an obstructive lung disease in primary care according to whether COPD is indicated by spirometry (FEV**_**1**_**/FVC <0.7; n = 149) or not (n = 227)**

	**FEV**_**1**_**/FVC <0.7**			**FEV**_**1**_**/FVC ≥0.7**		
	**OR**	**95% CI**	***P*-value**	**OR**	**95% CI**	***P*-value**
Age 65+ years	0.8	0.4–1.6	0.6	1.0	0.6–1.8	0.9
Male	0.6	0.4–1.3	0.7	1.0	0.6–1.8	0.9
Never smoker	1			1		
Current smoker	0.3	0.1–1.1	0.06	0.7	0.4–1.4	0.3
Previous smoker	0.5	0.1–1.4	0.5	0.7	0.4–1.3	0.3
Diagnosis (by GP)						
Asthma	3.1	1.6–6.2	0.001	6.3	1.4–28.4	0.02
COPD	0.5	0.2–1.2	0.1	0.6	0.3–1.2	0.1
Cardiovascular disease	0.4	0.2–0.8	0.01	1.2	0.7–2.2	0.6
Allergic illness	1.5	0.8–3.2	0.2	1.4	0.8–2.4	0.2
Exacerbation in the previous year	2.0	1.0–4.1	0.04	2.3	1.9–4.5	0.01
CCQ mean score^a^						
<1	1			1		
1–2	1.8	0.7–4.5	0.2	1.3	0.7–2.6	0.4
≥2	2.0	0.8–5.0	0.1	2.2	1.1–4.3	0.03
FEV_1_% predicted						
≥50%	1			1		
<50%	1.6	0.8–3.3	0.2	2.9	0.6–15.5	0.2

OR = odds ratio, CI = confidence interval.

^a^Data missing for 23 patients.

## Discussion

### Main findings

ICS/LABA was the most frequently prescribed drug in this study and was prescribed to 55.7% of the patients with COPD (FEV_1_/FVC <0.7), and the great majority of these patients had used this combination the day before the examination. ICS/LABA was prescribed more frequently than recommended in the 2007 GOLD guidelines for COPD patients without a concomitant asthma diagnosis, although a fair agreement between the recommendations and prescribing was found (Kappa = 0.32). Exacerbation in the previous year was a significant predictor among the COPD patients without a concomitant asthma diagnosis. However, this association was significant only when FEV_1_% predicted was <50 in the group only diagnosed with COPD . This explains why the actual ICS/LABA prescribing in 2009/10 was more consistent with the 2007–10 GOLD guidelines than with the later 2011 guidelines [1]. We also identified that concurrent cardiovascular disease was associated with decreased prescribing of ICS/LABA in COPD patients.

### Comparison with previous studies

Similar high prescription rates of ICS/LABA in COPD patients have been found in previous studies [5,6] and lower rates in others [12,13]. Although the use of ICS/LABA is recommended in subgroups of COPD patients, to our knowledge, no one has described the characteristics of patients who are prescribed this combination by their GP in real clinical practice. The importance attached to previous exacerbation by the GPs was also supported by the ECLIPSE study, which showed that previous exacerbation predicted future exacerbation [5], and by the TORCH study, which showed that ICS/LABA reduced the risk of exacerbation [14].

### Strengths and weaknesses

A strength of this study is that post-bronchodilator spirometry was performed and that 95.5% of the patients expired for six seconds or more [15]. Another strength of the study is the complete information on medication, symptoms and exacerbation provided by these dedicated GPs and patients. Although Norwegian GPs are usually well informed about the treatments given to their patients in secondary care, some underreporting regarding prescribed medication and hospitalizations may have occurred, but probably not in a systematic manner that would have affected the trends in our study. The inclusion of patients diagnosed with only asthma, only COPD, and patients with both diagnoses is another strength and reflects the diagnostic uncertainty in primary care. Some of the COPD patients with a concomitant asthma diagnosis probably had persistent asthma or asthma that was not optimally controlled, and some had a combination of asthma and COPD. Such patients are usually treated with ICS/LABA. Some of the patients who were registered as having asthma and who had an FEV_1_/FVC <0.7 were probably regular COPD patients. The shift in diagnostic labelling from asthma to COPD the past 15 years because of the new awareness of COPD as a separate diagnosis, is still progressing [15-17]. The Norwegian reimbursement regulations between 2006 and 2010, which restricted reimbursement of ICS/LABA costs to patients with asthma only, probably slowed this process. Because of the improper application of the asthma diagnosis among the COPD patients, the predictive value of asthma for ICS/LABA prescribing has probably been overestimated. A bigger problem in our analysis was the small size of the subgroup of patients with definitive COPD diagnosis (FEV_1_/FVC <0.7 and COPD as the only diagnosis given by the GP).

We did not register the frequency that patients had consulted their GP in the previous year. Patients with exacerbation in the previous year might have had a higher consulting rate than other patients and, accordingly, may have been more likely to have ICS/LABA prescribed. This might have led to an overestimation of previous exacerbation as predictor of such prescribing. On the other hand, a reduced exacerbation rate in patients because of treatment with ICS/LABA for more than 1 year might have weakened the association between exacerbation in the previous year and ICS/LABA prescribing.

The GP offices chose to participate in the study voluntarily and might not be representative of Norwegian practices. As shown in a previous paper, this probably did not affect the diagnostic labelling [15].

### Cardiovascular comorbidity and smoking

The GPs seemed to be more reluctant to prescribe ICS/LABA to COPD patients with cardiovascular comorbidity. The most recent guidelines have been criticized for not considering multimorbidity [18]. There has been too much emphasis on vertical integration across primary and secondary care and too little focus on holistic care to patients with multimorbidity [19]. In this critique COPD patients have been in focus because of the high prevalence of cardiovascular disease and depression in this disease group [20]. In the TORCH study, which evaluated the usefulness of ICS/LABA treatment in COPD, patients with cardiovascular comorbidity were not excluded, and 40% were on cardiovascular drugs [21]. Treatment with ICS/LABA was not associated with increased risk of adverse cardiovascular effects. Although cardiovascular comorbidity was not a criterion for exclusion in the TORCH study, patients likely to die of causes other than COPD in the coming three years were not included [21] and, for this reason, some patients with heart failure were probably excluded. Because ICS/LABA has not been proven to decrease mortality in COPD patients, an eagerness to add this medication might have been outweighed by concern about adverse effects and polypharmacy.

Current smoking was associated with decreased ICS/LABA prescribing among the COPD patients, although this finding was not significant and must be interpreted with caution. It is possible that some GPs postpone giving ICS/LABA treatment to smoking patients to maintain a strong focus on the most important measure for the patient, smoking cessation. GPs may wish to emphasize the patient’s responsibility for managing the medical condition and its future course.

### ICS/LABA for COPD in the future

The efficacy of ICS/LABA in COPD patients has been questioned in a recent Cochrane analysis [22,23], and discontinuation of such therapy in selected COPD patients has been shown to be safe [17,23]. Efforts to classify COPD patients into phenotypes may help ensure a more optimal use of ICS/LABA as maintenance treatment in COPD patients [24].

## Conclusion

Compared with the 2007 GOLD guidelines, ICS/LABA was overprescribed. The GPs seemed to emphasize previous exacerbation as a rationale in their prescribing but mainly for patients with severe COPD. The GPs were somewhat reluctant to prescribe ICS/LABA to COPD patients with cardiovascular comorbidity, possibly to avoid unnecessary adverse effects and polypharmacy.

## Competing interests

AØ has received speaker’s fees from GSK, BI, and Pfizer, and honorarium from advisory boards from BI.

## Authors’ contributions

ED: study preparation and execution, analyses, paper writing; AØ: study execution, analyses, paper writing; HM: study preparation, analyses, paper writing. All authors read and approved the final manuscript.

## Pre-publication history

The pre-publication history for this paper can be accessed here:

http://www.biomedcentral.com/1471-2296/15/42/prepub
